# Enhancing Whole Slide Image Classification in Renal Cell Carcinoma via Swin Transformer-Based Multiple Instance Learning

**DOI:** 10.3390/bioengineering13060680

**Published:** 2026-06-11

**Authors:** Bohan Zhang, Zhen Gao

**Affiliations:** Faculty of Engineering, McMaster University, Hamilton, ON L8S 4L8, Canada; zhanb6@mcmaster.ca

**Keywords:** renal cell carcinoma, whole slide images, multiple instance learning, CLAM, swin transformer, computational pathology, histopathology classification

## Abstract

Renal cell carcinoma (RCC) comprises histologic subtypes with distinct prognosis and treatment implications. This single-cohort study evaluated slide-level weakly supervised subtype classification for clear cell RCC (ccRCC), papillary RCC (pRCC), and chromophobe RCC (chRCC) using 928 diagnostic H&E whole-slide images (WSIs) from 928 patients in TCGA-RCC. We propose Swin-CLAM, a controlled modification of CLAM in which the conventional CNN patch encoder is replaced by an ImageNet-pretrained Swin-Tiny Transformer, while the CLAM-SB bag-level aggregation module is kept unchanged. WSIs were segmented, tiled into non-overlapping 256×256 patches at an effective 20× magnification, encoded offline, and classified using slide-level labels only. In five-fold patient-level cross-validation on TCGA-RCC, Swin-CLAM achieved a macro-averaged AUC of 0.976±0.008, an accuracy of 94.8±1.0%, and a macro-F1 of 0.940±0.012, with the largest gain observed for chRCC. Attention heatmaps and t-SNE plots were used as qualitative, exploratory analyses rather than formal evidence of interpretability. These results suggest that stronger patch-level representation can improve CLAM-based RCC subtype classification under a fixed MIL aggregator. However, the study does not establish clinical readiness, and external validation, calibration, domain-shift analysis, and expert region-level assessment are needed before practical deployment.

## 1. Introduction

Renal cell carcinoma (RCC) is a common malignant tumor of the kidney and includes several histologic subtypes with different molecular profiles, prognoses, and treatment implications [1,2,3,4]. The three major subtypes considered in this study are clear cell RCC (ccRCC), papillary RCC (pRCC), and chromophobe RCC (chRCC). ccRCC often shows nests or alveolar sheets of tumor cells with clear cytoplasm and a prominent delicate vascular network; pRCC is commonly characterized by papillary or tubulopapillary architecture and foamy macrophages; and chRCC typically exhibits pale eosinophilic or reticulated cytoplasm, distinct cell borders, and perinuclear halos [5]. These morphologic patterns support routine diagnosis, but RCC can be heterogeneous, and some pRCC and chRCC cases show overlapping cytoplasmic and architectural features that complicate subtype classification [3,5].

The widespread adoption of digital pathology and whole slide imaging (WSI) has created opportunities for computational tools that assist diagnosis, subtyping, prognosis, molecular prediction, and foundation-model representation learning from pathology images [6,7,8,9,10,11,12]. WSIs contain gigapixel-scale morphologic information but cannot usually be processed in a single forward pass. In addition, dense pixel-level or region-level annotations are expensive to obtain and difficult to standardize across institutions [7,13,14]. Weakly supervised multiple instance learning (MIL) addresses these constraints by representing each WSI as a bag of image patches and learning slide-level prediction models from slide-level labels only [14,15,16]. Attention-based MIL frameworks, including CLAM (Clustering-constrained Attention MIL), are widely used because they combine strong slide-level performance with attention maps that can be inspected qualitatively [14,15,17].

A common design choice in MIL pathology pipelines is to use a convolutional neural network (CNN), such as ResNet-50, as a patch-level encoder [14]. CNN features are effective for many histology tasks, but their local inductive bias may not capture all spatial configurations present in tumor nests, vascular patterns, stroma, and inflammatory infiltrates. This is particularly relevant for fine-grained subtype distinctions where cellular texture and local architecture jointly contribute to diagnosis. Vision Transformers and hierarchical variants such as Swin Transformer provide an alternative patch encoder by combining local window-based attention with hierarchical feature construction [18,19].

Transformer-based computational pathology models can be used at different levels of the WSI pipeline. TransMIL applies Transformer attention at the bag level to model interactions among patch embeddings [20], whereas HIPT is a hierarchical WSI Transformer framework that relies on multi-resolution self-supervised pretraining [21]. In contrast, the present study does not introduce a new MIL aggregator and does not attempt to build a full hierarchical WSI Transformer. Instead, it asks a narrower methodological question: when the bag-level CLAM module is held fixed, does replacing the patch encoder with Swin-Tiny improve RCC subtype classification?

The contributions of this study are therefore fourfold. First, we implement Swin-CLAM, a CLAM-SB-based MIL pipeline that replaces the conventional ResNet patch encoder with a frozen ImageNet-pretrained Swin-Tiny encoder. Second, we evaluate the model on slide-level TCGA-RCC subtype classification using patient-level five-fold cross-validation. Third, we compare Swin-CLAM against AB-MIL, ResNet-CLAM, and TransMIL under the same preprocessing and data-splitting protocol. Fourth, we report subtype-wise error patterns, fold-wise results, computational considerations, and qualitative visualization while explicitly acknowledging the limits of a single-cohort study.

## 2. Theoretical Framework

### 2.1. Weakly Supervised Learning on Whole Slide Images

Weakly supervised learning is well suited to WSI analysis because slide-level diagnostic labels are more readily available than dense annotations [7,14]. In the MIL formulation, a WSI is represented as a bag of instances, and a model learns to predict the slide label from the unordered set of patch features [14,15,16]. Simple pooling can be insufficient because only a subset of patches may contain diagnostic tumor regions, whereas other patches may contain stroma, necrosis, hemorrhage, benign kidney parenchyma, or artifacts.

Attention-based MIL addresses this limitation by assigning learnable importance weights to patches before aggregation [15]. CLAM extends attention-based MIL by adding an instance-level clustering constraint that encourages high-attention and low-attention instances to occupy separable regions in feature space [14]. Because CLAM is a strong, widely used baseline and provides a relatively modular separation between patch encoding and bag aggregation, it is suitable for isolating the effect of the patch encoder. In this study, CLAM-SB is kept unchanged, and the methodological contribution is the substitution of the patch-level encoder rather than a new attention or pooling mechanism.

For RCC, this controlled design is useful because subtype-discriminative information may appear at the level of local tissue architecture, cytoplasmic texture, and cell arrangement. If the aggregator remains fixed, performance differences between ResNet-CLAM and Swin-CLAM can be interpreted more directly as evidence that the patch representation affects downstream MIL performance. This interpretation remains associative rather than causal because additional factors, such as optimization noise and class imbalance, may still influence cross-validation results.

### 2.2. Transformer-Based MIL in Computational Pathology

Transformers have been incorporated into pathology MIL pipelines in several ways. TransMIL treats patch embeddings as tokens and applies Transformer attention at the bag level, thereby explicitly modeling inter-instance relationships among patches [20]. This differs from Swin-CLAM: Swin-CLAM uses Swin Transformer only to encode individual patches, and CLAM then aggregates the resulting patch embeddings as an unordered bag. Thus, Swin-CLAM should not be interpreted as directly modeling global WSI context in the same manner as a bag-level Transformer.

HIPT represents a different class of models. It uses a hierarchical image-pyramid design and self-supervised pretraining across multiple spatial scales to scale Transformer modeling to gigapixel WSIs [21]. Although such frameworks are powerful, they require substantial pretraining resources and a more complex multi-resolution pipeline. Our goal is more modest and modular: to test whether a readily available hierarchical vision Transformer backbone can strengthen patch features in an otherwise standard CLAM workflow.

Swin-Tiny was selected for this proof-of-concept study because it provides a favorable balance between representational capacity and computational cost. Its shifted-window attention captures interactions within local neighborhoods while maintaining manageable memory usage, and ImageNet-pretrained weights are publicly available. Larger Swin variants or histopathology foundation models may provide stronger representations, but using them would confound the controlled comparison and substantially increase computational requirements. We therefore treat histology-specific pretraining and foundation-model encoders as important future work rather than as part of the present experiment.

### 2.3. Computational Pathology for RCC

Computational pathology methods have investigated histologic classification, prognosis, molecular prediction, and multimodal fusion from WSIs [8,9,10,11,12,22]. Prior RCC-specific studies include weakly supervised grading systems for ccRCC and image models that incorporate nuclear or architectural features [23,24,25]. These studies support the premise that both local cellular morphology and larger tissue patterns are informative for renal tumor assessment.

The morphologic differences among ccRCC, pRCC, and chRCC provide a clinically meaningful motivation for subtype classification. ccRCC is often dominated by clear cytoplasm and a rich capillary network; pRCC frequently forms papillae or tubules and may contain macrophages or hemosiderin; chRCC often shows sharply defined cell borders, pale reticulated cytoplasm, and perinuclear clearing. However, fixation, sectioning, tumor heterogeneity, and variant morphology can obscure these patterns. The present study evaluates whether Swin-based patch features help a fixed CLAM aggregator separate these subtypes, with particular attention to pRCC–chRCC confusion.

## 3. Methodology

### 3.1. Problem Formulation and Overall Pipeline

We formulate RCC subtyping as slide-level three-class classification from H&E-stained WSIs. Let $D={\left\{\left(S^{\left(n\right)},y^{\left(n\right)}\right)\right\}}_{n=1}^{N}$ denote a dataset of *N* WSIs, where $S^{\left(n\right)}$ is the *n*-th slide and $y^{\left(n\right)}\in \left\{1,2,3\right\}$ denotes ccRCC, pRCC, or chRCC. Each WSI is decomposed into a bag of image patches:

(1) $$B^{\left(n\right)}={\left\{x_{i}^{\left(n\right)}\right\}}_{i=1}^{M_{n}},$$

where $x_{i}^{\left(n\right)}\in R^{H\times W\times 3}$ is a tissue patch and $M_{n}$ is the number of selected tissue patches for slide $S^{\left(n\right)}$.

Swin-CLAM contains two components. First, each patch is passed through a frozen Swin-Tiny encoder $f_{\mathrm{Swin}}\left(\cdot \right)$ to obtain a patch embedding:

(2) $$z_{i}^{\left(n\right)}=f_{\mathrm{Swin}}\left(x_{i}^{\left(n\right)}\right)\in R^{768}.$$

Second, the set of embeddings ${\left\{z_{i}^{\left(n\right)}\right\}}_{i=1}^{M_{n}}$ is used as input to a CLAM-SB aggregator, which computes attention scores and produces a slide-level subtype prediction. All primary metrics are reported at the slide level. When a patient had more than one eligible diagnostic slide, all slides from that patient were assigned to the same fold; patient-level exploratory predictions were obtained by averaging slide-level class probabilities, but the main tables report slide-level performance for consistency with the MIL training objective.

**Figure 1:**
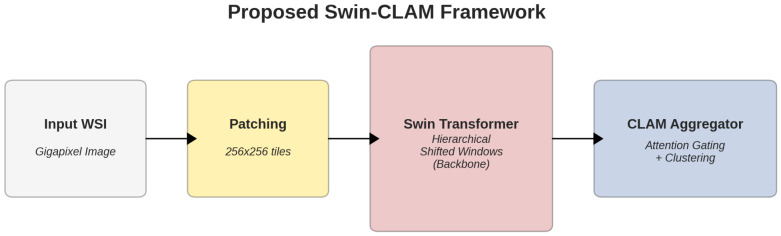
Overview of the Swin-CLAM workflow. Tissue regions are segmented, tiled into patches, encoded by a frozen Swin-Tiny patch backbone, and aggregated by an unchanged CLAM-SB module to obtain slide-level RCC subtype probabilities and attention heatmaps.

The overall pipeline is: (i) tissue segmentation, (ii) non-overlapping patch extraction at an effective 20× magnification, (iii) offline patch-level feature extraction with a frozen encoder, (iv) CLAM-SB training using slide-level labels, and (v) evaluation with cross-validation and qualitative attention visualization. Figure 1 summarizes the workflow.

### 3.2. Pre-Processing and Patch Extraction

WSIs were read with OpenSlide. The target resolution was an effective 20× magnification, corresponding approximately to 0.50μm/pixel when microns-per-pixel metadata were available. If the scanner metadata were incomplete, the objective-power field was used to estimate the down-sampling factor. Tissue segmentation was performed on a low-resolution thumbnail by converting the slide to HSV/grayscale space, applying Otsu thresholding, and using morphological closing and opening to remove small artifacts and fill holes.

Non-overlapping 256×256 pixel patches were extracted from tissue regions. A patch was retained only if at least 50% of its pixels belonged to the tissue mask and if fewer than 80% of pixels were near-white background pixels (RGB intensity greater than 220 in all channels). No stain normalization was applied in the reported experiments; this avoids introducing an additional preprocessing variable and makes the comparison focus on the feature backbone. Color normalization and stain-robust training are therefore treated as future work.

To keep memory and training time bounded, each slide bag was capped at $M_{\max}=5000$ patches. Slides with more than 5000 tissue patches were uniformly sampled without replacement after tissue filtering. In the final TCGA-RCC manifest, the median number of retained tissue patches per WSI was approximately 3740 (interquartile range: 2110–5000), with small slides contributing fewer patches and large slides reaching the cap.

### 3.3. Swin Transformer Backbone

We use Swin-Tiny as the patch-level feature extractor. Patches are extracted at 256×256 pixels and then resized to 224×224 pixels before feature extraction to match the ImageNet-pretrained Swin-Tiny input convention. The same resizing and ImageNet channel normalization were applied to ResNet-50 features used in the CNN-based baselines, so that the preprocessing conditions differed only in the encoder architecture.

The Swin-Tiny model was initialized with ImageNet-1K weights and used as a fully frozen encoder. We used the final-stage representation after global average pooling as a 768-dimensional patch embedding. Patch embeddings were computed offline and stored before MIL training. This design removes stochastic augmentation during MIL training; the reported results therefore do not rely on online patch augmentation after feature extraction. Fine-tuning was not performed because the goal was to isolate the effect of the backbone under a controlled and reproducible pipeline, and because end-to-end fine-tuning of large WSI bags would require substantially more GPU memory and may introduce additional hyperparameter sensitivity.

### 3.4. CLAM Aggregation Module

For bag-level aggregation, we used CLAM-SB rather than CLAM-MB. CLAM-SB was chosen because it is the most direct single-branch multiclass CLAM configuration and provides a clean controlled comparison when replacing the patch encoder. Given patch embeddings ${\left\{z_{i}\right\}}_{i=1}^{M}$, CLAM applies a gated attention network:

(3) $$a_{i}=\frac{\exp\;\left(w^{⊤}\left(\tanh\left(Vz_{i}^{⊤}\right)⊙\sigma \left(Uz_{i}^{⊤}\right)\right)\right)}{\sum_{j=1}^{M}\exp\;\left(w^{⊤}\left(\tanh\left(Vz_{j}^{⊤}\right)⊙\sigma \left(Uz_{j}^{⊤}\right)\right)\right)},$$

where *V*, *U*, and *w* are learnable parameters. The slide-level representation is the attention-weighted sum:

(4) $$z_{\mathrm{bag}}=\sum_{i=1}^{M}a_{i}z_{i}.$$

The CLAM-SB attention network used hidden dimensions of 512 and 256 with gated attention and dropout of 0.25. For the instance-level clustering branch, the top k=8 high-attention and bottom k=8 low-attention patches per slide were sampled for the positive and negative instance constraints, following the standard CLAM setting. Heatmaps were generated by mapping patch attention scores back to their WSI coordinates and min–max normalizing scores within each slide for visualization. Because attention weights are not equivalent to causal explanations, the heatmaps are interpreted only as qualitative indicators of regions influencing the model.

### 3.5. Loss Function and Optimization Objective

Given precomputed patch embeddings, the CLAM-SB aggregator is trained with a slide-level cross-entropy loss and an instance-level clustering loss:

(5) $$L_{\mathrm{total}}=c_{1}\;L_{\mathrm{CE}}+c_{2}\;L_{\mathrm{cluster}}.$$

We set $c_{1}=1.0$ and $c_{2}=0.1$ in the final experiments based on validation macro-AUC. The loss was computed per slide bag with batch size one bag, which is standard for WSI MIL training because bags have variable numbers of patches. Class imbalance was addressed through patient-level stratified folds and class-balanced slide sampling during training; no additional post hoc threshold tuning was applied. Early stopping used validation macro-AUC with a patience of 20 epochs, and the checkpoint with the highest validation macro-AUC was used for test evaluation.

## 4. Experiments

### 4.1. Dataset and Cohort Construction

We evaluated Swin-CLAM on TCGA-RCC, combining diagnostic H&E WSIs from TCGA-KIRC, TCGA-KIRP, and TCGA-KICH, the three major TCGA renal carcinoma projects summarized in the integrated TCGA-RCC analysis [3,4]. The final analyzed manifest contained 928 diagnostic WSIs from 928 patients: 519 ccRCC slides from TCGA-KIRC, 300 pRCC slides from TCGA-KIRP, and 109 chRCC slides from TCGA-KICH. We explicitly recorded the screening and exclusion criteria before model training. Slides/cases were excluded only if they lacked an unambiguous RCC subtype label, represented a non-diagnostic or nonrepresentative H&E record, could not be read reliably, or contained insufficient tissue after segmentation. All retained slides passed the tissue-content filter after background removal. In total, 963 candidate TCGA-RCC H&E records were screened; 35 cases/WSIs were excluded before model training, leaving 928 cases and 928 WSIs for analysis. The exact exclusion categories are reported in Table 1.

The final composition of the analyzed TCGA-RCC cohort is summarized in Table 2.

We used patient-level stratified five-fold cross-validation. In each run, 60% of patients were used for training, 20% for validation, and 20% for testing. When multiple slides belonged to a single patient, all slides were assigned to the same split; however, the final analyzed manifest contained one diagnostic slide per patient. The fold-by-fold class distribution is reported in Table 3 to make the split protocol explicit. Reported results are mean ± standard deviation over the five test folds unless otherwise specified.

### 4.2. Baselines

We compared Swin-CLAM with three weakly supervised WSI classification baselines. AB-MIL used the gated attention MIL formulation with ResNet-50 patch embeddings. ResNet-CLAM used the same CLAM-SB aggregator as Swin-CLAM but replaced Swin-Tiny with ImageNet-pretrained ResNet-50. TransMIL used a Transformer-based bag-level aggregator with the same ResNet-50 features as the CNN-based baselines. All baselines were retrained by the authors using the same slide splits, tissue segmentation, patch extraction, and evaluation protocol. Hyperparameters were selected on the validation split within each fold using the same macro-AUC criterion.

The comparison with TransMIL should be interpreted carefully. TransMIL changes the bag-level aggregation mechanism, whereas Swin-CLAM changes the patch-level encoder while keeping CLAM-SB fixed. Therefore, performance differences between Swin-CLAM and TransMIL cannot be attributed solely to global context modeling; they reflect a combination of different feature encoders and different aggregation strategies.

### 4.3. Training and Implementation Details

Patch features were precomputed offline for all methods. For Swin-CLAM, each 256×256 tissue patch was resized to 224×224 and encoded by a frozen ImageNet-1K Swin-Tiny backbone, producing a 768-dimensional vector. For ResNet-based methods, the same patch coordinates and resizing were used, and the final pooled ResNet-50 feature vector was stored. Because embeddings were precomputed, no online image augmentation was used during MIL training in the reported experiments.

The CLAM-SB and AB-MIL models were trained with Adam, learning rate $2\times 10^{-4}$, weight decay $1\times 10^{-5}$, batch size one slide bag, and a maximum of 100 epochs. TransMIL used the same optimizer and validation-based early stopping. The random seeds for the five folds were 1, 2, 3, 4, and 5. All experiments were run on a single NVIDIA GPU with 24 GB memory. Offline feature extraction was the most expensive step; after feature extraction, training one Swin-CLAM fold required approximately 2–3 h, depending on the number of capped bags. Table 4 provides a practical computational summary.

### 4.4. Evaluation Metrics

For each fold, we computed macro-averaged one-vs-rest AUC, overall accuracy, macro-F1, and subtype-wise precision, sensitivity, specificity, and F1-score. Confusion matrices were computed from pooled test predictions across folds. Statistical comparisons between Swin-CLAM and the strongest baselines used paired fold-level macro-AUC values with a Wilcoxon signed-rank test. Because the sample size for statistical testing is only five folds, the resulting *p* values are reported as descriptive evidence rather than definitive proof.

## 5. Results and Analysis

### 5.1. Quantitative Performance

Table 5 summarizes the overall cross-validation results. Swin-CLAM achieved the highest mean macro-AUC, accuracy, and macro-F1 among the compared methods. The improvement over ResNet-CLAM was consistent across the primary metrics, supporting the hypothesis that the patch encoder influences CLAM-based WSI classification. The improvement over TransMIL was smaller. In paired fold-level macro-AUC comparisons, Swin-CLAM was higher than both ResNet-CLAM and TransMIL in all five folds; however, with only five folds, two-sided exact Wilcoxon signed-rank tests gave p=0.063 for both comparisons. We therefore report these tests as descriptive evidence and interpret the baseline comparisons cautiously rather than claiming definitive superiority.

### 5.2. Subtype-Wise Performance and chRCC Analysis

**Figure 2:**
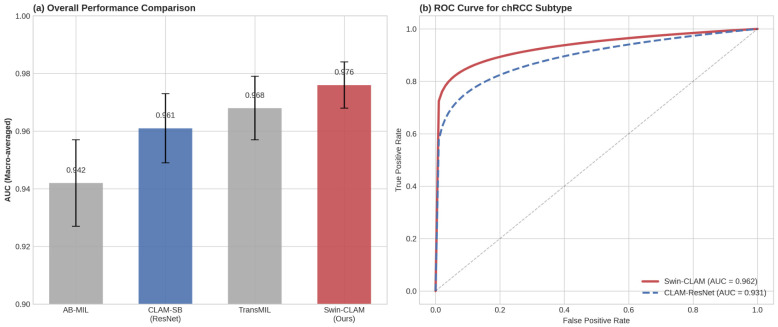
Overall performance comparison on TCGA-RCC. (**a**) Overall performance comparison using macro-averaged AUC for AB-MIL, ResNet-CLAM, TransMIL, and Swin-CLAM, with error bars indicating cross-validation variability. (**b**) One-vs-rest ROC curves for chRCC comparing ResNet-CLAM and Swin-CLAM.

**Figure 3:**
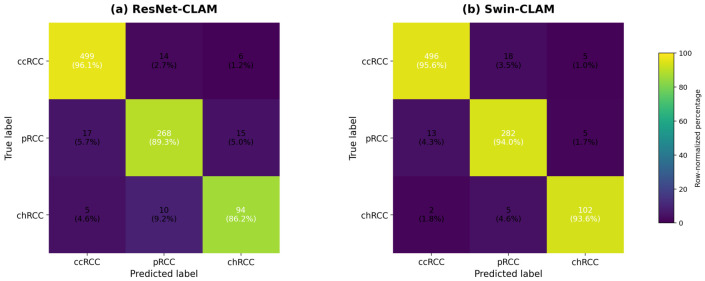
Pooled confusion matrices for ResNet-CLAM and Swin-CLAM. Each cell shows the absolute number of slides and the row-normalized percentage. Swin-CLAM reduces pRCC–chRCC confusion, although errors remain in both directions.

The overall performance comparison and chRCC ROC analysis are shown in Figure 2. Both methods performed best on ccRCC, which is the largest subtype and often has more distinctive clear-cell morphology. The main gain from Swin-CLAM was observed in the minority chRCC class, where sensitivity and F1-score increased relative to ResNet-CLAM. This is consistent with the reduced pRCC–chRCC confusion shown in Figure 3.

Fold-wise macro-AUC values are listed in Table 6 to show the cross-validation variability of each method.

Subtype-wise metrics for ResNet-CLAM and Swin-CLAM are reported in Table 7.

The error analysis indicates that the most clinically relevant residual errors occur between pRCC and chRCC. This pattern is plausible because both subtypes can present eosinophilic cytoplasm and variable architecture in selected regions. Swin-CLAM reduces but does not eliminate this confusion, suggesting that stronger patch representations help but cannot fully replace expert review, multi-scale context, or ancillary clinical and molecular information.

### 5.3. Feature Space Visualization

**Figure 4:**
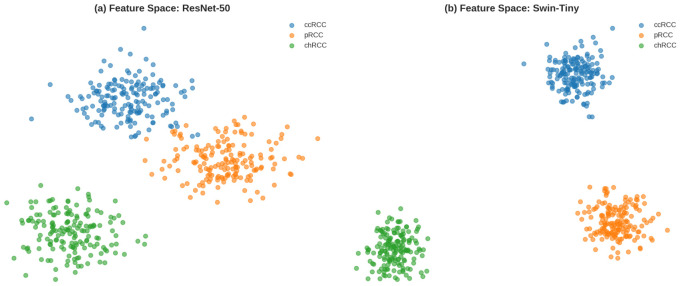
t-SNE visualization of slide-level embeddings for ResNet-CLAM and Swin-CLAM. Points are colored by subtype. The plot is used only as exploratory evidence because t-SNE does not provide a formal statistical test of representation quality.

We performed t-SNE on slide-level representations obtained after MIL aggregation. For each model, t-SNE was fitted separately on the same pooled test slides using perplexity 30, learning rate 200, 1000 iterations, and random seed 1. The visualization used all test-slide embeddings pooled across the cross-validation folds. Figure 4 suggests more compact clustering for Swin-CLAM, particularly for chRCC. However, t-SNE is sensitive to initialization and hyperparameters and should be interpreted as exploratory visualization rather than quantitative proof of class separability.

### 5.4. Qualitative Attention and Interpretability

Attention heatmaps were inspected qualitatively for representative slides from each subtype. In many ccRCC slides, both ResNet-CLAM and Swin-CLAM assigned high attention to tumor-rich regions. For pRCC and chRCC, Swin-CLAM more often concentrated attention on regions showing papillary architecture or chromophobe-like cytoplasmic features, whereas ResNet-CLAM more frequently highlighted mixed tumor–stroma areas or visually nonspecific tissue. These observations are compatible with the quantitative results but are not a formal validation of interpretability.

We therefore treat attention maps as a tool for model inspection rather than as evidence that the model has learned pathologist-equivalent reasoning. Rigorous validation would require reader studies, region-level annotations, perturbation-based testing, or comparison with pathologist-marked diagnostic regions. Such validation was outside the scope of the present TCGA-only experiment.

## 6. Discussion

### 6.1. The Role of Feature Representation in MIL

The results support the view that patch-level representation is an important determinant of MIL performance in WSI classification. Because ResNet-CLAM and Swin-CLAM use the same CLAM-SB aggregator, the comparison provides a controlled test of the encoder substitution. Swin-CLAM showed higher mean macro-AUC and macro-F1 than ResNet-CLAM, with the strongest relative improvement for chRCC. This finding is consistent with the expectation that a hierarchical Transformer patch encoder can encode tissue patterns that complement CLAM attention.

At the same time, the study should not be read as proving that Swin-CLAM captures global WSI organization better than all alternatives. In our implementation, Swin operates at the patch level, while the MIL aggregator still treats patch embeddings as a bag. Spatial coordinates are used for heatmap reconstruction but not explicitly modeled by CLAM-SB. Therefore, any benefit from Swin should be described as improved within-patch and local-context representation rather than as direct global WSI context modeling.

### 6.2. Handling Histological Heterogeneity in RCC

RCC subtype classification is affected by tumor heterogeneity, variable tissue quality, and overlapping morphology. The reduced pRCC–chRCC confusion in Swin-CLAM suggests that the backbone may better preserve cytoplasmic and architectural cues relevant to this distinction. Nevertheless, residual errors remain, particularly for minority classes. These results indicate that patch-level feature improvement can help but is not sufficient for a clinically complete solution.

A practical diagnostic system would also need calibration, uncertainty estimates, robust rejection of low-confidence cases, and validation across scanners, stains, and institutions. The current model does not include those components. Its intended contribution is methodological: evaluating whether a Swin patch encoder is a useful substitution inside a controlled CLAM pipeline.

### 6.3. Limitations and Future Directions

This study has several limitations. First, the evaluation is limited to TCGA-RCC, and no independent external cohort was used. TCGA slides come from multiple contributing centers, but they do not replace prospective, multi-institutional validation. Scanner variability, stain differences, and local tissue-processing protocols may affect performance under domain shift.

Second, the cohort is imbalanced, with chRCC being the smallest class. Although stratified splitting and class-balanced sampling were used, minority-class estimates remain less stable than ccRCC estimates. Third, the model was trained for slide-level classification only and was not calibrated. Calibration, uncertainty estimation, and confidence-aware triage are essential for clinical decision support. Fourth, the attention maps and t-SNE plots are qualitative exploratory tools; they require region-level evaluation or reader studies before interpretability claims can be made.

Fifth, formal ablation studies were not performed in the present work beyond the controlled comparison between ResNet-CLAM and Swin-CLAM. We did not separately ablate Swin variant size, encoder fine-tuning, patch size, magnification, stain normalization, CLAM-SB versus CLAM-MB, or the top-*k* instance-clustering setting. These analyses are planned as future work, preferably together with an external validation cohort so that ablation conclusions can be tested under domain shift.

Sixth, the Swin-Tiny backbone was pretrained on ImageNet and kept frozen. This choice supports a controlled comparison and reduces computational cost, but it leaves a domain gap between natural images and histopathology. Future work should evaluate histology-pretrained encoders, self-supervised pretraining on RCC slides, and pathology foundation models. Finally, Swin-based feature extraction is moderately more expensive than ResNet-based extraction. Lightweight Transformers, model distillation, or patch-selection strategies may be needed for large-scale deployment.

### 6.4. Potential Extensions

Future studies should test Swin-CLAM on independent RCC cohorts and across institutions, scanner vendors, and staining protocols. Multi-scale modeling may also be useful because RCC diagnosis involves both cytologic details and architectural context beyond a single 20× patch. In addition, integrating clinical variables, radiologic findings, or genomic features could make the model more relevant to real-world RCC workflows, where histology is interpreted together with other patient information [3,22].

## 7. Conclusions

We presented Swin-CLAM, a weakly supervised RCC subtype classification pipeline that replaces the conventional ResNet patch encoder in CLAM-SB with a frozen Swin-Tiny Transformer while leaving the MIL aggregator unchanged. On slide-level five-fold cross-validation using TCGA-RCC, Swin-CLAM achieved higher mean macro-AUC, accuracy, and macro-F1 than the evaluated baselines, with the most notable improvement for chRCC.

These findings suggest that strengthening patch-level representation can improve CLAM-based RCC subtyping under a controlled experimental setup. The conclusion is limited to the TCGA-RCC cohort and should not be interpreted as evidence of clinical readiness. External validation, domain-shift testing, calibration, uncertainty estimation, and quantitative assessment of attention maps remain necessary before considering clinical translation.

## Figures and Tables

**Table 1:** Screening and exclusion log for TCGA-RCC cohort construction. Cases and WSIs are reported separately so that duplicate or multi-slide patients can be identified when applicable.

Screening Category	Cases	WSIs
Candidate TCGA-RCC H&E records screened	963	963
Retained in final analysis	928	928
Excluded before model training, total	35	35
Missing or ambiguous subtype metadata	10	10
Non-diagnostic or nonrepresentative H&E record	15	15
Unreadable or corrupted image file	0	0
Insufficient tissue after segmentation	10	10

**Table 2:** Final TCGA-RCC cohort used for slide-level analysis after screening.

Subtype	TCGA Project	Patients	WSIs
ccRCC	TCGA-KIRC	519	519
pRCC	TCGA-KIRP	300	300
chRCC	TCGA-KICH	109	109
Total	–	928	928

**Table 3:** Fold-by-fold slide/case distribution used for five-fold patient-level cross-validation. Values are reported as ccRCC/pRCC/chRCC (total). Because the analyzed manifest contains one slide per patient, slide and case counts are identical.

Run	Training Set	Validation Set	Test Set
Fold 1	311/180/65 (556)	104/60/22 (186)	104/60/22 (186)
Fold 2	311/180/65 (556)	104/60/22 (186)	104/60/22 (186)
Fold 3	311/180/65 (556)	104/60/22 (186)	104/60/22 (186)
Fold 4	312/180/66 (558)	103/60/21 (184)	104/60/22 (186)
Fold 5	312/180/66 (558)	104/60/22 (186)	103/60/21 (184)

**Table 4:** Approximate computational characteristics under the reported implementation. Times are intended to indicate practical scale rather than hardware-independent benchmarks.

Method	Encoder Parameters	Feature Dim.	Feature Extraction	MIL Training/Fold
ResNet-CLAM	25.6M	2048	Faster	1–2 h
TransMIL	25.6M + aggregator	2048	Faster	2–3 h
Swin-CLAM	28.3M	768	Moderate	2–3 h

**Table 5:** Overall slide-level RCC subtype classification performance on TCGA-RCC. Values are mean ± standard deviation across five patient-level test folds.

Method	Encoder/Aggregator	Macro-AUC	Accuracy	Macro-F1
AB-MIL	ResNet-50/Attn-MIL	0.942±0.015	0.903±0.019	0.883±0.024
ResNet-CLAM	ResNet-50/CLAM-SB	0.961±0.012	0.928±0.015	0.912±0.019
TransMIL	ResNet-50/TransMIL	0.968±0.011	0.939±0.014	0.925±0.016
Swin-CLAM (ours)	Swin-Tiny/CLAM-SB	0.976±0.008	0.948±0.010	0.940±0.012

**Table 6:** Fold-wise slide-level macro-AUC. The table is included to make cross-validation variability explicit.

Method	Fold 1	Fold 2	Fold 3	Fold 4	Fold 5
AB-MIL	0.928	0.957	0.936	0.958	0.931
ResNet-CLAM	0.948	0.972	0.955	0.973	0.957
TransMIL	0.957	0.981	0.963	0.975	0.964
Swin-CLAM	0.969	0.982	0.971	0.986	0.974

**Table 7:** Subtype-wise slide-level metrics from pooled test predictions across five folds.

Method	Subtype	Precision	Sensitivity	Specificity	F1-Score
ResNet-CLAM	ccRCC	0.958	0.962	0.946	0.960
ResNet-CLAM	pRCC	0.918	0.893	0.962	0.905
ResNet-CLAM	chRCC	0.817	0.862	0.974	0.839
Swin-CLAM	ccRCC	0.971	0.956	0.963	0.963
Swin-CLAM	pRCC	0.925	0.940	0.963	0.932
Swin-CLAM	chRCC	0.911	0.936	0.988	0.923

## Data Availability

The data analyzed in this study are publicly available from The Cancer Genome Atlas (TCGA) through the Genomic Data Commons Data Portal at https://portal.gdc.cancer.gov/, specifically from the TCGA-KIRC, TCGA-KIRP, and TCGA-KICH projects. No new datasets were generated in this study.
